# Metabolic Composition and Quality Traits of *Polygonatum cyrtonema* Hua from Different Germplasms and Age Sections Based on Widely Targeted Metabolomics Analysis

**DOI:** 10.3390/ijms24076077

**Published:** 2023-03-23

**Authors:** Qingshuang Wang, Jingjie Ban, Roudi Cai, Xueying Zhang, Chunwang Lai, Yan Chen, Xiaoli Li, Cuirong Chen, Yukun Chen, Zihao Zhang, Zhongxiong Lai, Yuling Lin

**Affiliations:** Institute of Horticultural Biotechnology, Fujian Agriculture and Forestry University, Fuzhou 350002, China

**Keywords:** *Polygonatum cyrtonema* Hua, germplasm, age section, widely targeted metabolite, differential metabolites

## Abstract

*Polygonatum* rhizomes are rich in various compounds with many biological activities and are widely used in functional foods and pharmaceutical products. In order to screen for superior *Polygonatum cyrtonema* Hua (*P. cyrtonema*) germplasm and also to elucidate the nutritional and medicinal values of rhizomes, the metabolic composition and quality traits of rhizomes from different germplasms and age sections of *P. cyrtonema* were analysed by widely targeted metabolomics, and the molecular mechanism of triacylglycerol synthesis was explored. The results showed that the different germplasms and age sections of *P. cyrtonema* were rich in different nutritional and medicinal components. Of these, the broad-leaved green stem (GK) germplasm is rich in polysaccharides, alkaloids, and lipids; the pointed-leaved green stem (JL) germplasm is rich in flavonoids, steroids, and amino acids, while the pointed-leaved purple stem (JZ) germplasm contains more phenolic acids. The one-year (AT) age section is rich in polysaccharides, steroids, organic acids, and lipids; the three years (CT) age section contains more flavonoids, alkaloids, and amino acid metabolites. Lipids were significantly enriched in the broad-leaved green stem germplasm and the one-year age section. Interestingly, the highest accumulation of triacylglycerols, an important component of lipids, was also found in the GK germplasm and the AT age section. Nineteen, 14, and 13 members of the *glycerol-3-phosphate acyltransferase* (*GPAT*), *lysophosphatidic acid acyltransferase* (*LPAT*), and *diacylglycerol acyltransferase* (*DGAT*) gene families, respectively, involved in triacylglycerol synthesis were also identified. The quantitative real-time PCR (qRT-PCR) results further suggested that the differentially expressed *PcDGAT1*, *PcDGAT2.4*, *PcGPAT9.1*, *PcLPAT2.9*, and *PcLPAT4.3* genes may play important roles in triacylglycerol synthesis in *P. cyrtonema*. Therefore, this study provides a new theoretical reference for product development and the breeding of new varieties of *Polygonatum* species.

## 1. Introduction

The genus *Polygonatum* is a perennial herb of the Liliaceae family that is distributed worldwide, including in China, Russia, Korea, Japan, India, Europe, and North America [1]. *Polygonatum* species germplasm resources, including *Polygonatum cyrtonema* Hua (*P. cyrtonema*), *Polygonatum kingianum* Coll. et Hemsl, and *Polygonatum sibiricum* Red et al., are abundant in China, and *Polygonatum* species are naturally distributed in Fujian, Anhui, Zhejiang, and Jiangxi Provinces. Among these species, *P. cyrtonema,* including pointed-leaved green stem, pointed-leaved purple stem, broad-leaved green stem, and broad-leaved purple-green germplasm, is a key constituent of herbal medicines in Fujian Province and is mainly distributed near the Wuyi Mountains. The rhizome contains a variety of beneficial substances, such as polysaccharides, steroidal saponins, and flavonoids, which exert hypoglycaemic, antioxidant, anticancer, antidepressant, antibacterial, and anti-inflammatory effects [2,3,4,5]. *P. cyrtonema*, as one of the three medicinal *Polygonatum* species, is included in the “Pharmacopoeia of the People’s Republic of China” (Edition 2020). Moreover, *P. cyrtonema* can be processed into flour and dried fruit and preserves, which are very popular with consumers. In addition, *P. cyrtonema* plants are beautiful and can be planted in flower beds, forests, and other locations to enhance the environment. Thus, *P. cyrtonema* is an economic plant with edible, medicinal, and ornamental value and broad prospects for exploitation. Varietal characteristics are a key factor in determining the yield and quality of cultivated *P. cyrtonema* [6]. Related studies have suggested that differences in the metabolic composition between varieties of the same species may result in species with different qualities and characteristics [7,8]. Some variation in the medicinal composition of *Polygonatum* species from different germplasms has been reported [9,10], and significant differences in the medicinal composition of *Polygonatum* species from the same germplasms have been found among different age sections and varying cultivation environments [11]. In recent years, the contents of total polysaccharides, total saponins, and some medicinal components, such as total flavonoids, have primarily been used as criteria to evaluate the quality of germplasms and age sections of *Polygonatum* species. However, *Polygonatum* species contain a variety of chemical components, such as lipids, alkaloids, and phenolic acids, in addition to polysaccharides, steroids, and flavonoids. Therefore, traditional methods for the analysis of some medicinal components, such as total polysaccharides, total saponins, and total flavonoids, do not allow a detailed evaluation of *P. cyrtonema* germplasms and age sections.

Plant metabolomics describes the metabolic networks of organisms and their mechanisms of action at the metabolite level and can be used to corroborate or guide the accuracy of plant classification [12]. Widely targeted metabolomics analysis is a technology that combines the advantages of untargeted and targeted metabolite assays, which allows comparison of the metabolite species and abundance between samples, the screening for biologically significant metabolites between groups, and the accurate characterisation of metabolites with high throughput, high sensitivity, and broad coverage [13,14]. Widely targeted metabolomics has been extensively used in the identification and analysis of many plant metabolites, such as the analysis of differences in the medicinal compositions of different varieties of *Curcuma* [8], the exploration of differences in the nutritional compositions of black and white sesame (*Sesamum indicum* L.) seeds [15] and the analysis of metabolic differences among different coloured radishes (*Raphanus sativus* L.) [7]. A previous study of the genus *Polygonatum* revealed that adenosine, sucrose, and pyroglutamic acid could be used as markers to distinguish three *Polygonatum* species (*Polygonatum kingianum* Coll. et Hemsl, *Polygonatum cyrtonema* Hua, and *Polygonatum sibiricum* Red) by ultra-performance liquid chromatography/quadrupole time-of-flight tandem mass spectrometry (UPLC-Q-TOF-MS/MS) metabolomics [9]. In addition, the nutritional and medicinal components of different types of rhizomes of *P. cyrtonema* and *Polygonatum sibiricum* Red have been explored based on high-performance liquid chromatography coupled with charged aerosol detection (HPLC–CAD) and ultrahigh-performance liquid chromatography-Orbitrap-tandem mass spectrometry (UHPLC-Orbitrap-MS/MS) metabolomics [10]. However, a widely targeted metabolomics analysis of *P. cyrtonema* has not been reported in the literature.

Lipids are important metabolic components in plants and play an important role in plants. Moreover, Triacylglycerol (TAG) is a major lipid and an important carbon and energy reserve. Studies have shown that environmental stresses can induce the accumulation of TAG in nutritive plant tissues. TAG induces the synthesis of lipid droplets and plastoglobules, which are important for plant stress resistance [16]. In addition, studies have shown that the *GPAT*, *LPAT*, and *DGAT* genes are key genes in the TAG synthesis pathway [17]. TAG is closely related to plant stress resistance; thus, investigating the mechanism of TAG synthesis could provide a new theoretical basis for research into plant stress resistance.

In recent years, the *P. cyrtonema* industry has developed rapidly due to expanding market demand. However, there is a lack of extensive studies on the metabolite levels in *P. cyrtonema*, and elucidating the differences in metabolites between different germplasms and age sections of *P. cyrtonema* is particularly important for providing a scientific basis for the harvesting, processing, grading and classification, and rational clinical use of *P. cyrtonema*. Preliminary research has revealed that three germplasms, JL, JZ, and GK, are the main cultivated germplasms near the Wuyi Mountains. In this study, different metabolites from these three *P. cyrtonema* germplasms (JL, JZ, and GK) and three age sections (one year (AT), two years (BT), and three years (CT)) of the GK germplasm were analysed by widely targeted metabolomics. The differences in the nutritional and medicinal composition between the different germplasms and age sections of *P. cyrtonema* were clarified. In addition, this study analysed the TAG content and related synthetic genes in the three germplasms and three age sections and speculated on the key genes involved in TAG synthesis. The results of this study provide a new theoretical basis and a reference for the breeding and product development of *Polygonatum* species varieties.

## 2. Results

### 2.1. Phenotypic Variations among Different P. cyrtonema Germplasms

**Figure 1:**
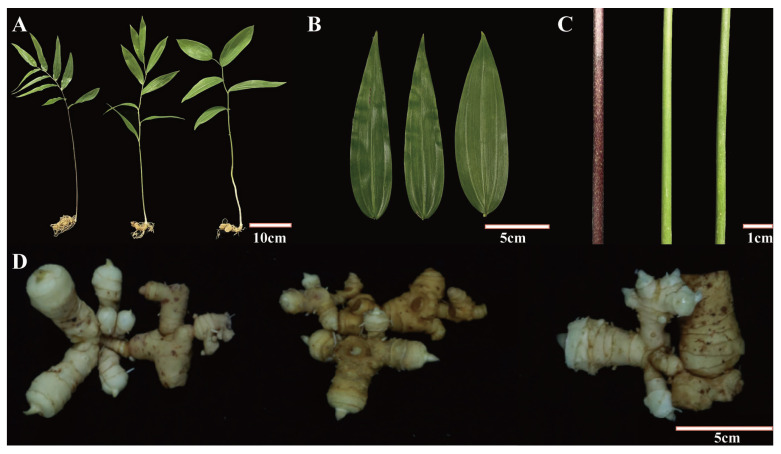
Phenotypic characteristics of different *P. cyrtonema* germplasm. (**A**–**D**): From left to right, JZ, JL, and GK germplasms.

The three *P. cyrtonema* germplasms used in this study, JL, JZ, and GK, are the main cultivated germplasms in the Wuyi Mountains, China. Under consistent growing conditions, the JL germplasm has narrow, slender leaves and green stems; the JZ germplasm has narrow, slender leaves and purple stems; and the GK germplasm has broad leaves and green stems (Figure 1A–C). The rhizomes of all three *P. cyrtonema* germplasms were of the ginger type (similar to ginger) and did not significantly differ (Figure 1D), but significant differences in both the leaf shape and stem colour were observed among the three germplasms, indicating possible variations in the metabolic characteristics of the germplasms. Based on these results, a widely targeted metabolomics analysis was performed to characterise the metabolic differences among the three germplasms.

### 2.2. Widely Targeted Metabolomic Analysis of Rhizomes from Different Germplasms of P. cyrtonema

**Figure 2:**
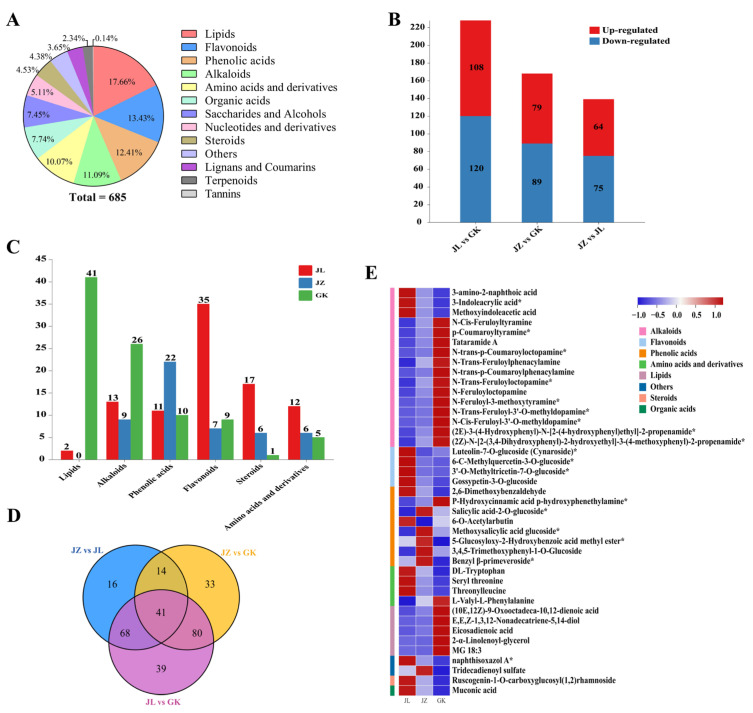
Widely targeted metabolomic analysis of rhizomes from JL, JZ, and GK germplasms in *P. cyrtonema*. (**A**) Metabolite classification. (**B**) Up-regulated and down-regulated differential metabolites. Red boxes represent up-regulation, and blue boxes represent down-regulation. (**C**) The relative amounts of the highest differential metabolites in the three germplasms. Red boxes represent JL germplasm, blue boxes represent JZ germplasm, and green boxes represent GK germplasm. (**D**) Venn diagram of differential metabolites. (**E**) Heat map of 41 key differential metabolites, “*” indicates an isomer.

To better understand the metabolic differences between rhizomes from different germplasms of *P. cyrtonema* from the Wuyi Mountains, a widely targeted metabolomics analysis of one-year-old rhizomes from the JL, JZ and GK germplasms was performed. A total of 685 metabolites in 13 classes were identified, including 121 lipids, 92 flavonoids, 85 phenolic acids, 76 alkaloids, 69 amino acids and their derivatives, 53 organic acids, 51 saccharides and alcohols, 35 nucleotides and their derivatives, 31 steroids, 30 other classes of metabolites, 25 lignans and coumarins, 16 terpenoids, and one tannin (Figure 2A).

The samples were analysed by PCA, OPLS-DA, and HCA to discriminate the magnitudes of the variation between and within groups of samples from the JL, JZ, and GK germplasms. PCA revealed a trend of separation among the three groups of samples (JZ, JL, and GK), showing a trend of separation on PC1, and the cumulative contribution of PC1 and PC2 is 40.85% (Figure S2A). The OPLS-DA of the samples (Figure S3A–C) indicated a significant effect of differentiation within the three groups of samples, JL vs. GK, JZ vs. GK, and JZ vs. JL. The indicators in the evaluation parameters of the OPLS-DA model had indicator values greater than 0.4 and Q^2^ > 0.7. The OPLS-DA model was well constructed and could be used to screen differential metabolites based on their VIP values. The peak areas of each metabolite were then log_10_ transformed and subjected to HCA (Figure S2B). This study found that the three germplasms (JL, JZ, and GK) were significantly different, with clustering of the JL and JZ germplasms suggesting that the JL and JZ germplasms are more closely related. Furthermore, the PCA, OPLS-DA, and HCA results indicated that the three germplasms had distinct metabolite profiles.

The three *P. cyrtonema* germplasms (JL, JZ, and GK) were subjected to pairwise comparisons, and a total of 291 differential metabolites in 13 classes, mainly flavonoids, alkaloids, phenolic acids, lipids, steroids, and amino acids and derivatives, were identified in the three groups. Specifically, 228 differential (120 downregulated and 108 upregulated) metabolites were identified from the JL vs. GK comparison; 168 differential (89 downregulated and 79 upregulated) metabolites were identified from the JZ vs. GK comparison; and 139 differential (75 downregulated and 64 upregulated) metabolites were identified from the JZ vs. JL comparison (Figure 2B). The differential metabolites obtained from the three comparisons (JL vs. GK, JZ vs. GK, and JZ vs. JL) could be divided into 12, 12, and 13 different classes, and the primary metabolites among these differential metabolites were mainly lipids. Notably, almost all lipid metabolites were upregulated in the GK germplasms compared with the JL and JZ germplasms. The three sets of differential metabolites (identified from the JL vs. GK, JZ vs. GK, and JZ vs. JL comparisons) were plotted on a Venn diagram (Figure 2D), and 41 key differential metabolites were screened in eight categories (Table S2), including 16 alkaloids, eight phenolic acids, five lipids, four flavonoids, four amino acids and their derivatives, two other classes, one steroid, and one organic acid. The highest number of specific differential metabolites was obtained from the JL vs. GK comparison (39), followed by the JZ vs. GK comparison (33), and the lower number was identified from the JZ vs. JL comparison (16). These findings indicate that the metabolites of the three *P. cyrtonema* germplasms differ greatly and that metabolite, such as alkaloids, phenolic acids, lipids, flavonoids, and amino acids, are probably the main components responsible for the differences between the three *P. cyrtonema* germplasms.

Interestingly, a comparison of the relative contents of the major differential metabolites of the three germplasms revealed that 41 lipids (95.35% of the total number of differential lipid metabolites) and 26 alkaloids (54.17% of the total number of differential alkaloid metabolites) had the highest relative contents in the GK germplasm; 35 flavonoids (68.63%), 17 sterols (70.83%), and 12 amino acids and their derivatives (52.17%) were present at significantly higher relative amounts in the JL germplasm than in the GK and JZ germplasms; and 22 phenolic acids (51.16%) were present in the JZ germplasm (Figure 2C). The results show that the GK germplasm is rich in lipids and alkaloids; the JL germplasm is rich in flavonoids, steroids, and amino acids and their derivatives; and the JZ germplasm contains more phenolic acids. Lipids not only form the structural basis of biological membranes but also play an important role in the plant body as signalling molecules and energy sources [18]. Notably, almost all of the differential lipid-like metabolites (95.35%) were found in the highest relative amounts in the GK germplasm, and KEGG enrichment analyses of the three germplasms were further performed to probe the metabolic processes of lipids.

### 2.3. KEGG Enrichment Analysis to Determine the Differences in Lipid Metabolites among Rhizomes from Different Germplasms of P. cyrtonema

**Figure 3:**
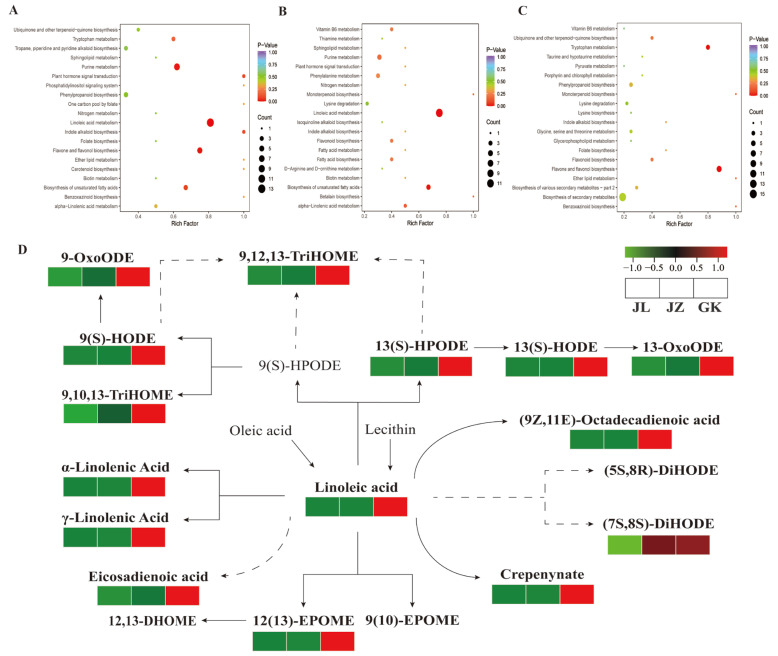
(**A**–**C**) KEGG enrichment plots of differential metabolites from different germplasms comparison groups, (**A**) JL vs. GK, (**B**) JZ vs. GK, (**C**) JZ vs. JL, (**D**) Distribution of differential metabolites from different germplasms *P. cyrtonema* linoleic acid and unsaturated fatty acid biosynthetic pathways, grey font represents non-detected metabolites, solid arrows represent individual reactions, and the dashed arrows indicate conversions with multiple steps catalysed.

A KEGG enrichment analysis of the differential metabolites identified from pairwise comparisons of three *P. cyrtonema* germplasms (JL, JZ, and GK) was performed according to the KEGG classifications of the differential metabolite. Sixty-four, 38 and 37 differential metabolites identified from the JL vs. GK, JZ vs. GK, and JZ vs. JL comparisons, respectively, were annotated to the corresponding metabolic pathways, respectively. The differential metabolites screened were enriched in a total of 77 metabolic pathways, mainly linoleic acid metabolism (ko00591), flavonoid and flavonol biosynthesis (ko00944), purine metabolism (ko00230), unsaturated fatty acid biosynthesis (ko01040), and tryptophan metabolism pathways (ko00380) (*p* < 0.05) (Figure 3A–C). Among these pathways, the biosynthetic pathways of linoleic acid metabolism and unsaturated fatty acids are closely related to lipid metabolism, and the biosynthetic pathways of linoleic acid metabolism and unsaturated fatty acids were mapped to the three germplasms based on available reports and metabolic pathway databases (Figure 3D). Linoleic acid metabolism was significantly enriched in the metabolites identified from the JL vs. GK and JZ vs. GK comparison. Thirteen differential lipid metabolites identified from the JL vs. GK comparison, including crepenynic acid, γ-linolenic acid, and linoleic acid, exhibited significantly higher accumulation in the GK germplasm than in the JL germplasm (FC > 2.99); 12 differential lipid metabolites identified from the JZ vs. GK comparison related to crepenynic acid, γ-linolenic acid, and linoleic acid showed significantly higher accumulation in the GK germplasm than in the JZ germplasm (ranging from 2.65 to 23.02 times). Unsaturated fatty acid biosynthesis was significantly enriched in the metabolites identified from JZ vs. GK comparison and involved four differential metabolites, namely γ-linolenic acid (FC = 23.02), α-linolenic acid (FC = 16.75), linoleic acid (FC = 16.32), and eicosadienoic acid (FC = 8.37). Thus, the KEGG enrichment analysis results further validate the finding that the GK germplasm contains the highest amount of lipid metabolites.

### 2.4. Widely Targeted Metabolomics Analysis of Rhizome Sections of Different Ages Sections of P. cyrtonema

**Figure 4:**
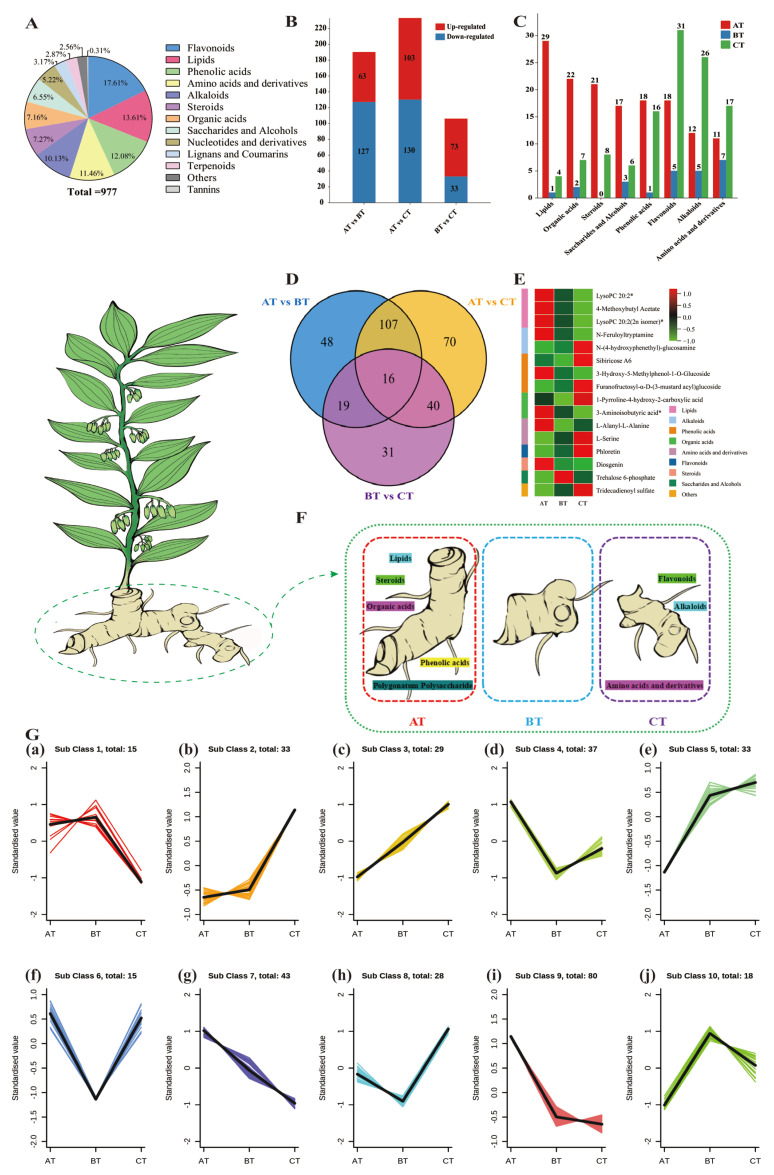
Widely targeted metabolomic analysis of rhizome sections of different ages in GK germplasm. (**A**) Metabolite classification. (**B**) Up- and down-regulated differential metabolites. Red boxes represent up-regulation, the blue boxes represent down-regulation. (**C**) Relative amounts of the highest differential metabolites in the three age classes. Red boxes represent annual age classes, blue boxes represent second-year age classes, and green boxes represent third-year age classes. (**D**) Venn diagram of differential metabolites. (**E**) Heat map of 16 key differential metabolites, “*” indicates an isomer. (**F**) Different age sections have rich metabolite classes. (**G**) K-means clustering analysis of differential metabolites in the ten subclasses.

The rhizomes of the GK germplasm contain significantly more lipids and alkaloids than those of the JL and JZ germplasms, which may be more advantageous for food and medicinal purposes. Therefore, this study further assessed the metabolites of rhizome sections from different age sections of the GK germplasm by widely targeted metabolomics analysis. A total of 977 metabolites were identified and divided into 13 different categories (Figure 4A). Among these, primary metabolites (430), secondary metabolites (522), and other categories (25) accounted for 44.01%, 53.43%, and 2.56% of the total number of metabolites, respectively. The primary metabolites included lipids (133 species, 13.61%), amino acids and their derivatives (112 species, 11.46%), organic acids (70 species, 7.16%), saccharides and alcohols (64 species, 6.55%), and nucleotides and their derivatives (51 species, 5.22%); the secondary metabolites included flavonoids (172 species, 17.61%), phenolic acids (118 species, 12.08%), alkaloids (99 species, 10.13%), sterols (71 species, 7.27%), lignans and coumarins (31 species, 3.17%), terpenoids (28 species, 2.87%), and tannins (three species, 0.31%).

The samples were analysed by PCA and HCA, which showed a clear trend of separation and significant differences between the three groups of samples (AT, BT, and CT), indicating that the different age sections had varying metabolic profiles (Figure S2C,D). Of these, the cumulative contribution of PCA1 and PCA2 was 47.19%. The OPLS-DA showed that the samples in the AT vs. BT, AT vs. CT, and BT vs. CT comparisons were clearly differentiated internally (Figure S3D–F), and the OPLS-DA model was well constructed because all the evaluation parameters of the OPLS-DA model had indicator values greater than 0.5 and Q^2^ > 0.8.

A total of 331 differential metabolites were identified from the AT vs. BT, AT vs. CT, and BT vs. CT comparisons. Of these, 190 differential (127 downregulated and 63 upregulated) metabolites were identified from the AT vs. BT comparison, and these mainly included alkaloids, flavonoids, organic acids, saccharides and alcohols, amino acids and their derivatives, phenolic acids, steroids, and lipids. In addition, 233 differential (130 downregulated and 103 upregulated) metabolites, which mainly included alkaloids, lipids, phenolic acids, organic acids, amino acids and their derivatives, flavonoids, and saccharides and alcohols, were identified in AT vs. CT comparison, and 106 differential (33 downregulated and 73 upregulated) metabolites, which mainly comprised flavonoids and alkaloids, were detected from the BT vs. CT comparison (Figure 4B). Venn diagrams of the three sets of differential metabolites (those obtained from the AT vs. BT, AT vs. CT, and BT vs. CT comparisons) were plotted, and 9/16 classes sharing differential metabolites were further screened (Figure 4D,E, Table S3). These nine classes of differential metabolites included three lipids, three phenolic acids, two alkaloids, two amino acids and their derivatives, two organic acids, one flavonoid, one steroid, one sugar and alcohol, and one other class (Figure 4E), which could be used as markers to distinguish between one-, two-, and three-year sections of rhizomes.

Notably, a comparison of the relative contents of the main differential metabolites in the three age sections (AT, BT, and CT) revealed that 29 lipids (85.29%), 22 organic acids (70.97%), 21 sterols (72.41%), and 17 saccharides and alcohols (65.38%) had the highest relative contents in the AT age section; 31 flavonoids (57.41%), 26 alkaloids (60.47%), and 17 amino acids and their derivatives (48.57%) were present at the highest relative content in the CT age section. Furthermore, this study revealed that 18 (51.43%) and 16 (45.71%) phenolic acids had the highest relative contents in the AT and CT age sections, respectively (Figure 4C). Thus, the AT age section is richer in lipids, organic acids, steroids, saccharides and alcohols than the other age sections; the CT age section is richer in flavonoids, alkaloids, and amino acids and their derivatives than the other age sections; and the AT and CT age sections are richer in phenolic acids than the BT age section (Figure 4F).

To determine trends in differential metabolites in *P. cyrtonema* rhizome sections of different ages, the relative levels of all differential metabolites were z-score normalised and subsequently subjected to K-means cluster analysis to divide the differential metabolites into ten subclasses (Figure 4G). Subclasses 1 through 10 contained 15, 33, 29, 37, 33, 15, 43, 28, 80, and 18 metabolites, respectively. A total of 37.16% (123 species) of the differential metabolites showed a gradual decrease in metabolite content with increasing age, and 28.7% (95 species) of the differential metabolites showed a gradual increase in metabolite content with increasing age. Among the subclasses, subclass 9 contained the highest number of differential metabolites (24.17%, 80 species). The metabolite content of this subclass in the rhizome sections decreased sharply from 1 to 2 years of age and decreased more slowly from 2 to 3 years of age (Figure 4G(i)). Furthermore, subclass 7 contained 43 differential metabolites, mainly lipids (15 species, 34.88%), which showed decreasing metabolite accumulation with increasing age (Figure 4G(g)). In addition, subclass 3 included 29 differential metabolites, such as sinapic acid (SA), the metabolite content of which gradually accumulated with increasing age (Figure 4G(c)).

### 2.5. KEGG Enrichment Analysis of Differential Metabolites of P. cyrtonema Rhizome Sections of Different Ages

**Figure 5:**
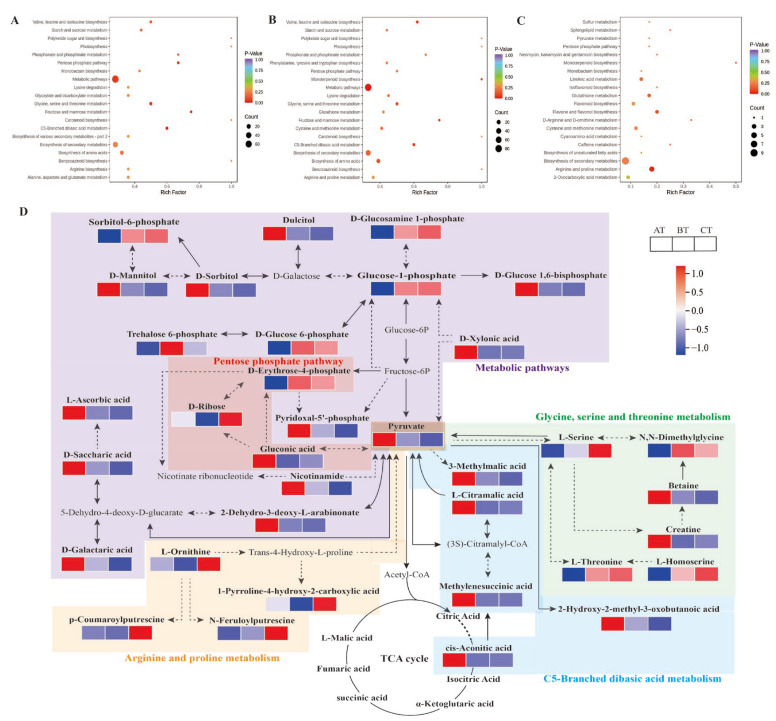
(**A**–**C**) Differential metabolite KEGG enrichment maps and (**D**) Metabolic pathway maps for different age sections of *P. cyrtonema,* grey font represents non-detected metabolites, solid arrows represent individual reactions, and the dashed arrows indicate conversions with multiple steps catalysed. (**A**) AT vs. BT, (**B**) AT vs. CT, (**C**) BT vs. CT.

This study performed KEGG differential metabolite enrichment analyses of three age sections of *P. cyrtonema* (AT, BT, and CT). Ninety-nine, 112 and 25 differential metabolites identified from the AT vs. BT, AT vs. CT, and BT vs. CT comparisons, respectively, were annotated to the corresponding metabolic pathways. The differential metabolites screened with significant enrichment (*p* < 0.05) were enriched in a total of 81 metabolic pathways, mainly including C5-diacid metabolism (ko00660), the pentose phosphate pathway (ko00030), arginine and proline metabolism (ko00330), metabolic pathways (ko01100), and glycine, serine and threonine metabolic (ko00260) pathways (Figure 5A–C).

To gain a comprehensive understanding of the variations in *P. cyrtonema* metabolites across age sections, this study constructed a network of the major differential metabolic pathways across the three age sections based on the literature and metabolic pathway databases (Figure 5D). The metabolite composition of *P. cyrtonema* rhizomes shows dynamic changes with age and contains many complex pathways. The metabolic and pentose phosphate pathways mainly involve sugar alcohol metabolites, most of which were found at the highest relative levels in the one-year age section (AT) and showed a decreasing trend with increasing age. Sugars, the main energy substances for plant growth and development, are broken down through the glycolytic pathway to produce pyruvate, which is involved in C5-diacid metabolism and glycine, serine and threonine metabolic pathways. Interestingly, this study showed that the organic acid content in the rhizome sections decreased with increasing age, which is in line with the trend found for the pyruvate content, whereas the amino acid group showed a tendency to accumulate in the rhizome sections with increasing age, in contrast with the trend found for the pyruvate content. Therefore, these results suggest that differences in the pentose phosphate pathway (ko00030) and metabolic pathways (ko01100) may be the main reasons for the differences in *P. cyrtonema* metabolites in the rhizome sections of different age sections.

### 2.6. Determination of the Triacylglycerol and Polysaccharide Contents of Rhizomes of Different Germplasms and Age Sections and Analysis of Triacylglycerol Synthesis-Related Genes in P. cyrtonema

**Figure 6:**
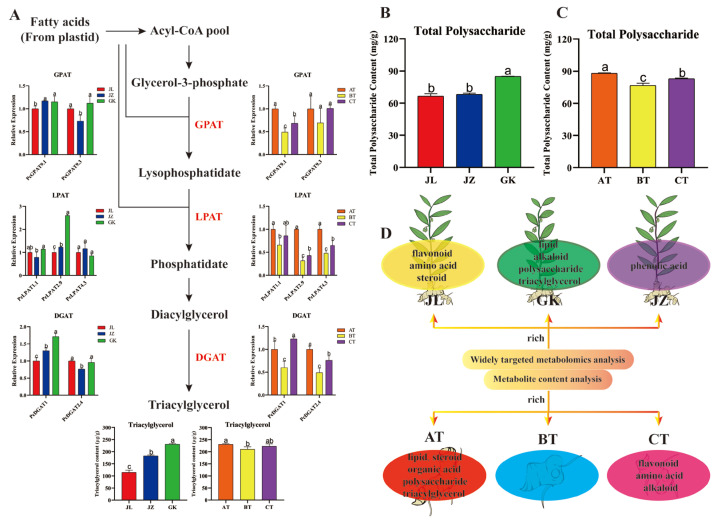
Analysis of triacylglycerol synthesis pathways and metabolic components in *P. cyrtonema*. (**A**) Triacylglycerol content and triacylglycerol biosynthesis pathway of different germplasms and different age sections, (**B**) Total polysaccharide content of different germplasms, (**C**) Total polysaccharide content of different age sections, (**D**) Enrichment map of the main nutritional and medicinal components of *P. cyrtonema* in different germplasms and different age sections. The lowercase letters (a, b, and c) indicate significant difference (*p* < 0.05).

Previous studies have found that TAGs are the main energy reserve in the form of lipids and play an important role in resisting the stress from adversity [16]. In this study, a total of 10 glycerides were identified in rhizomes from the three germplasms (JL, JZ, and GK). Four of these metabolites were differential glycerides and expressed at the highest levels in the GK germplasm. Therefore, these findings indicate that the GK germplasm may contain higher levels of TAG. Subsequently, this study measured the TAG contents in the three germplasms (JL, JZ, and GK) and detected a significantly higher level in the GK germplasm (230.87 μg/g) than in the JL (115.31 μg/g) and JZ germplasms (182.80 μg/g) (Figure 6A), suggesting that the GK germplasm may exhibit greater resistance to stress due to its higher TAG content. Interestingly, among the GK germplasm rhizome sections of different ages (AT, BT, and CT), the highest TAG content (230.87 μg/g) was observed in the AT age section, followed by the CT age section (223.2 μg/g), and the lowest was found in the BT age section (210.93 μg/g) (Figure 6A). Thus, the AT age section could be used in production as a material for the asexual propagation of *Polygonatum* species.

To investigate the molecular mechanisms of TAG synthesis in the three *P. cyrtonema* germplasms, transcriptome data from different tissues of *P. cyrtonema* were analysed. A total of 19, 14, and 13 members in the *GPAT*, *LPAT*, and *DGAT* gene families were identified, respectively. Seven members (*DGAT1*, *DGAT2.4*, *GPAT9.1*, *GPAT9.3*, *LPAT1.1*, *LPAT2.9*, and *LPAT4.3*) with high relative expression and differential expression in rhizomes were then selected for qRT-PCR analysis (Table S4). The qRT-PCR results showed that the *PcDGAT1* and *PcLPAT2.9* genes were significantly more highly expressed in the rhizomes of the GK germplasm and expressed at the lowest levels in the JL germplasm; additionally, the *PcDGAT2.4*, *PcGPAT9.1*, *PcLPAT2.9*, and *PcLPAT4.3* genes were significantly more highly expressed in the AT age section and expressed at the lowest levels in the BT age section. The expression of the abovementioned genes was consistent with the trend found for the TAG content, suggesting that these genes may be key genes involved in TAG synthesis in *P. cyrtonema* (Figure 6A).

Moreover, polysaccharides are the main active constituents in medicinal *Polygonatum* species. Fewer sugars were detected in this study, probably due to the higher throughput of the widely targeted metabolomics analysis and the greater interference of ions in the mass spectra. Therefore, this study determined the polysaccharide contents in the rhizomes of the three germplasms (JL, JZ, and GK) and three age sections (AT, BT, and CT) of *P. cyrtonema*. The results showed that the polysaccharide content of the GK germplasm (84.86 mg/g) was significantly higher than that of the JL (66.58 mg/g) and JZ germplasms (68.23 mg/g) (Figure 6B); the polysaccharide content of the AT age section (88.12 mg/g) was significantly higher than that of the BT (76.73 mg/g) and CT age sections (82.96 mg/g) (Figure 6C). The above results suggest that the GK germplasm and the AT age section may have higher polysaccharide-related nutritional and medicinal values.

## 3. Discussion

In recent years, widely targeted metabolomics has been utilised for metabolite analysis and species identification in a variety of plants, including loquat (*Eriobotrya japonica* Lindl) [19], chestnut (*Castanea mollissima* Bl.) [20], and ‘Hupingzao’ jujube (*Ziziphus jujuba* Mill.) [21]. Although researchers have reported many metabolic components in *Polygonatum* species, previous studies have focused on only a few metabolites, such as polysaccharides, saponins, and flavonoids [9,10]. The metabolite differences in rhizomes among different germplasms and different age sections of *P. cyrtonema* have not yet been studied in depth. This study constituted the first widely targeted metabolomics analysis of *P. cyrtonema* and identified a greater number of metabolite components than in previous studies.

### 3.1. Three P. cyrtonema Germplasms with Different Nutritional and Medicinal Components

In this study, a total of 685 metabolites in 13 classes and 291 differential metabolites were identified in the three *P. cyrtonema* germplasms (JL, JZ, and GK). A total of 228, 168, and 139 differential metabolites were identified from the JL vs. GK, JZ vs. GK, and JZ vs. JL comparisons, respectively, indicating the existence of different metabolic profiles among the different *P. cyrtonema* germplasms. Among the differential metabolites, the majority of lipids and alkaloids were the most abundant in the GK germplasm; flavonoids, steroids, and amino acids and their derivatives exhibited the highest levels in the JL germplasm; and phenolic acids were the most abundant in the JZ germplasm (Figure 6D). Lipids are a complex group of biomolecules that not only provide organisms with the necessary energy but also act as signalling molecules to regulate the organism’s vital activities. For example, ω-3 fatty acids can alleviate high-fat food-induced type 2 diabetes by inhibiting NLRP3 inflammatory vesicles and reducing the secretion of IL-1b, a key factor in inflammation [22]. TAG produces glycerol and free fatty acids through enzymatic hydrolysis reactions, and both of these products provide energy to the body through phosphorylation and β-oxidative catabolism, respectively [23]. In addition, previous studies have revealed low alkaloid levels in *P. cyrtonema* [24], but this study found a variety of alkaloids with medicinal activity in *P. cyrtonema*. For example, trigonelline and agmatine exert importance in hypoglycaemic, anticancer, antibacterial, and antidepressant effects [25,26]. Thus, due to its high levels of lipids and alkaloids, the GK germplasm shows potential for development and medicinal use. *P. cyrtonema* contains important active ingredients, such as flavonoids and steroidal saponins, with pharmacological activities, such as hypoglycaemic, antitumour, anti-inflammatory, antibacterial, and immune-modulating activities, similar to other medicinal plants [27,28]. The type and content of amino acids are key indicators of nutritional quality and an important determinant of flavour [19,29]. In this study, the JL germplasm was found to be richer in flavonoid, steroid, and amino acid metabolites. Therefore, the JL germplasm has better medicinal value in terms of flavonoids and steroids and greater nutritional value in terms of amino acids. As an important secondary metabolite in plants, phenolic acids play important roles in anti-inflammatory, antibacterial, antioxidant, and antitumour activities [30]. The JZ germplasm is rich in phenolic acids and has greater medicinal value in terms of phenolic acids than the other two germplasms (Figure 2C).

It is worth noting that polysaccharides are the main active components of medicinal *Polygonatum* species and one of the main bases for the evaluation of medicinal *Polygonatum* species, as stipulated in the 2020 edition of the Pharmacopoeia of the People’s Republic of China. Polysaccharides play important roles in immunomodulation, diabetes prevention and control, and antioxidant, antitumour, anti-inflammatory, antibacterial, and antidepressant activities [4]. In this study, the total polysaccharide contents of the three germplasms were determined by the anthrone-sulfuric acid method, which revealed that the GK germplasm contained significantly higher levels of polysaccharides than the JL and JZ germplasms (Figure 6D) and that the GK germplasm had higher medicinal and nutritional values in terms of polysaccharides. The study conducted by Li et al. revealed that one-year rhizomes of *P. cyrtonema* have a polysaccharide content of 7.76% (77.6 mg/g) [31], which was lower than that found in the GK germplasm in this study (84.86 mg/g) but higher than levels in the JL (66.58 mg/g) and JZ (68.23 mg/g) germplasms. Thus, it appears that the polysaccharide content varies among different germplasms of *P. cyrtonema*. However, some researchers found that the total polysaccharide content of purple stem *P. cyrtonema* was higher than green stem [32], which is not consistent with the results of this study. This may be due to germplasm differences in *P. cyrtonema* or different growth environments.

In summary, the three *P. cyrtonema* germplasms have different nutritional and medicinal components, and these characteristics may provide a reference for the rational development of medicinal metabolites from medicinal plants.

### 3.2. One-Year and Three-Year P. cyrtonema Rhizomes Have Higher Functional Value

The differences in the metabolic compositions of medicinal plants directly affect the quality of herbal medicines, and the compositions and contents of the active ingredients of medicinal plants are influenced by the number of years of cultivation [33]. To further understand the metabolic differences between the different age sections of *P. cyrtonema*, the three age sections of GK germplasms were analysed by widely targeted metabolomics analysis, which resulted in the identification of 977 metabolites in 13 classes and 331 differential metabolites. The results revealed some variations in the metabolite contents between the different age sections of *P. cyrtonema*. A comparison of the relative contents of the main differential metabolites revealed that the majority of lipids, organic acids, sterols, saccharides and alcohols were found at the highest relative contents in the AT age section (Figure 6D). It is possible that these substances accumulate in large quantities in the AT age section to provide the energy needed for plant growth and development. Organic acids are valuable due to their involvement in anti-inflammatory, antioxidant, antithrombotic, and defensive mechanisms against cardiovascular disease [34]. A previous study demonstrated that the total polysaccharide content of *P. cyrtonema* is highest in the one-year age section, followed by the three-year age section, and lowest in the two-year age section, which is consistent with the results of the present study (Figure 6C) [35]. However, in other studies, the polysaccharide content was observed to decrease gradually with increasing age [36], which is inconsistent with the results of the present study. One potential reason for this discrepancy is that the varying growth environments and different sampling times resulted in differences in the polysaccharide contents of *P. cyrtonema* rhizomes of different age sections. In addition, the majority of flavonoids, alkaloids, and amino acids and their derivatives were detected at the highest relative amounts in the CT age section (Figure 6D). Most of the phenolic acids were found at the highest relative levels in the AT and CT age sections. The results of this study provide a basis for the exploitation of different age sections of plants.

### 3.3. Broad-Leaved Green Stem Germplasm and One-Year-Old P. cyrtonema Sections May Exhibit Higher Resistance

This study found that 95.35% of the differential lipid-like metabolites accumulated at the highest levels in the GK germplasm. A KEGG enrichment analysis revealed that the differential metabolites were significantly enriched in the linoleic acid metabolism and unsaturated fatty acid biosynthesis pathways, which are closely related to lipid metabolism, and all the differential lipid metabolites involved in the pathways exhibited the highest accumulation in the GK germplasm, further confirming that the GK germplasm is rich in lipid metabolites. Glycerol ester and its related metabolites are major components of cell membranes and are part of the dynamic signalling processes associated with plant growth and development and resistance to biotic and abiotic stresses [37]. Environmental stresses such as temperature stress, nutrient deficiencies and high light usually result in the accumulation of TAG in nutritional tissues to help plants withstand adversity [38,39]. Four differential glyceride metabolites (1-α-linolenic acid glycerides, 2-α-linolenic acid glycerides, 1-linoleoyl glycerides, and 2-linoleoyl glycerides) were identified in the three germplasms and found at the highest levels in the GK germplasm, and the TAG content was also highest in the GK germplasm (Figure 6D), suggesting that the GK germplasm may exhibit higher resistance to stress.

It has been shown that *TmDGAT1* overexpression promotes the accumulation of TAG in garden nasturtium (*Tropaeolum majus*) [40], whereas *AhLPAT2* gene overexpression has an important role in TAG synthesis in peanut (*Arachis hypogaea*) [41]. In this study, a qRT-PCR analysis of TAG synthesis-related genes revealed that the *PcDGAT1* and *PcLPAT2.9* genes were significantly overexpressed in the GK germplasm, whereas the *PcDGAT2.4*, *PcGPAT9.1*, *PcLPAT2.9*, and *PcLPAT4.3* genes were significantly overexpressed in the AT age section of the GK germplasm. The trends found for the abovementioned genes were consistent with the trends found for the TAG content across germplasms and age sections. This study, therefore, hypothesised that the *PcDGAT1*, *PcDGAT2.4*, *PcGPAT9.1*, *PcLPAT2.9*, and *PcLPAT4.3* genes might be key genes for TAG synthesis in *P. cyrtonema* and, thus, lays a foundation for the screening and breeding of resistant germplasms of *Polygonatum* species.

## 4. Materials and Methods

### 4.1. Plant Materials

All *P. cyrtonema* test materials were collected from the *P. cyrtonema* Good Germplasm Breeding Base in Shaowu, Nanping City, Fujian Province. Three *P. cyrtonema* germplasms (JL, JZ, and GK) with 3- to 5-year-old rhizomes were harvested in September 2021, and the AT age section of rhizomes was used for related studies. The materials for all three age sections (AT, BT, and CT) of rhizomes were obtained from the GK germplasm and harvested in October 2021. The harvested materials were all 3- to 5-year-old rhizomes. A total of 3 randomly selected plants were mixed from each material, and the experiment was biologically replicated three times.

### 4.2. Sample Pretreatment

*P. cyrtonema* samples were freeze-dried using a vacuum freeze-dryer (Scientz-100F, Labconco, Kansas City, MO, USA). The samples were then ground (30 Hz, 1.5 min) to powder form using a mixer mill (MM 400, Retsch, Haan, Germany). A total of 100 mg of lyophilised powder was dissolved in 1.2 mL of 70% methanol solution, vortexed for 30 s every 30 min (six times in total), and placed the sample in a refrigerator at 4 °C overnight. After centrifugation at 12,000 rpm for 10 min, the supernatants were aspirated, and the samples were filtered through a microporous membrane (pore size of 0.22 μm). The samples were stored in injection vials for ultra-performance liquid chromatography-tandem mass spectrometry (UPLC–MS/MS) analysis. Some rhizome samples were dried to a constant weight in an electric blast-drying oven (BPG-9140A, Shanghai Yiheng, Shanghai, China), then ground to powder with a mortar and pestle and used for polysaccharide content determination. The remaining rhizome samples were snap-frozen in liquid nitrogen and stored at −80 °C for determination of the TAG content and analysis of gene expression.

### 4.3. UPLC-MS/MS Analysis

The analytical conditions for UPLC (Nexera X2, Shimadzu, Kyoto, Japan) were as follows: the chromatogram was an Agilent SB-C18 (1.8 µm, 2.1 mm × 100 mm), and the mobile phase consisted of solvent A, pure water with 0.1% formic acid, and solvent B, acetonitrile with 0.1% formic acid. Sample measurements were performed using a gradient program with starting conditions of 95% A and 5% B. Within 9 min, a linear gradient to 5% A and 95% B was achieved, and a composition of 5% A and 95% B was maintained for 1 min. Subsequently, the composition of the mobile phase was adjusted to 95% A and 5.0% B within 1.1 min, and the resulting composition was maintained for 2.9 min. The column temperature was set to 40 °C, the injection volume was 4 μL, and the flow rate was 0.35 mL/min.

The MS analysis conditions were as follows: linear ion trap (LIT) and triple quadrupole (QQQ) scans were acquired using a triple quadrupole-linear ion trap mass spectrometer (Q TRAP, AB Sciex, Framingham, MA, USA). AB4500 Q TRAP UPLC/MS/MS system equipped with an ESI Turbo Ion-Spray interface. The MS analysis was performed using electrospray ionisation (ESI) at a temperature of 550 °C and mass spectral voltage of 5500 V with a curtain gas (CUR) pressure of 25 psi and high collision-activated dissociation (CAD). The obtained data were processed using the Analyst 1.6.3 software (AB Sciex, Framingham, MA, USA).

### 4.4. Triacylglycerol and Polysaccharide Content Analysis

The TAG content of the *P. cyrtonema* samples was assayed using a triacylglycerol content assay kit (Comin Biotechnology, Suzhou, China) according to the kit instructions.

Anhydrous glucose (analytically pure) was used as the standard in reference to the anthraquinone-sulfuric acid method described in the 2020 edition of the Pharmacopoeia of the People’s Republic of China (I) [42]. The absorbance at 582 nm was measured with a multifunctional enzyme marker (infinite M200 PRO, Tecan, Switzerland Confederation). Using the degree of absorption as the vertical coordinate and the anhydrous glucose content as the horizontal coordinate, the following regression curve was obtained: *y =* 0.0104 *+* 19.872*x*, *r*^2^ = 0.994 (*n* = 6). The polysaccharide content was determined as described above.

### 4.5. Triacylglycerol Synthesis-Related Gene Screening Based on Transcriptome Data

In this study, because genomic data for *P. cyrtonema* have not yet been published, a transcriptome database (unpublished) of different tissue parts of *P. cyrtonema* (fruits, leaves, stems, roots, and rhizomes) was analysed, and the sequences of the *GPAT*, *LPAT*, and *DGAT* family genes and amino acids from *Arabidopsis thaliana* were downloaded from the TAIR database (https://www.arabidopsis.org/, accessed on 9 July 2022). The transcriptome was extracted from five parts (fruit, leaves, stalks, roots, and rhizomes), and cDNA libraries were constructed. The cDNA libraries were sequenced using the Illumina Hiseq high-throughput sequencing platform and filtered by fastp (0.19.3) software [43] to obtain 105.1 Gb of clean date. Subsequently, the clean date was assembled using Trinity (v2.11.0) software [44] to obtain 300,667 transcripts, of which Unigene included 277,955.

To identify the members of the *GPAT*, *LPAT*, and *DGAT* gene families in *P. cyrtonema*, the known *GPAT*, *LPAT*, and *DGAT* protein sequences of *Arabidopsis thaliana* were used as query sequences. Using reference transcriptome data from different tissue sites of *P. cyrtonema,* local BLAST (TBtools v1.098769, Guangzhou, China) [45] comparative searches were performed to initially screen for members of the *GPAT*, *LPAT*, and *DGAT* families in *P. cyrtonema*. The candidate sequences were submitted to the NCBI Batch Web CD-Search Tool (https://www.ncbi.nlm.nih.gov/Structure/bwrpsb/bwrpsb.cgi, accessed on 9 July 2022) and HMMER (https://www.ebi.ac.uk/Tools/hmmer/, accessed on 10 July 2022) for further identification and screening of the conserved domains. After removing sequences with incomplete domains and redundancies, members of the *P. cyrtonema GPAT*, *LPAT*, and *DGAT* gene families were obtained.

### 4.6. Quantitative Real-Time PCR (qRT-PCR) Analysis

The qRT-PCR primers for the genes were designed using the online website Primer 3 Input (https://primer3.ut.ee/, accessed on 14 September 2022) [46] and DNAMAN software (Table S1) [47]. Total RNA was extracted using the RNAprep Pure Plant Plus Kit (Polysaccharides & Polyphenolics-rich) RNAprep Pure Kit (TIANGEN, Beijing, China). The RNA quality was analysed by agarose gel electrophoresis (Figure S1B,D) and quantified using a Nanodrop 2000 spectrophotometer (Thermo Scientific, Wilmington, DE, USA). Only RNAs that met the criteria 260/280 ratio of 1.8–2.1 and 260/230 ratio ≥2.0 were used for further analyses.

The first strand cDNA was synthesised by reverse transcription using the PerfectStart^®^ Uni RT&qPCR Kit (TransGen Biotech, Beijing, China). The synthesised cDNA was diluted 10-fold, and the qRT-PCRs were performed on a Roche LightCycler96 (LightCycler96, Roche, Basel, Switzerland) instrument using SYBR Green chemistry (Hieff qPCR SYBR Green Master Mix, Yeasen, Shanghai, China). The melting curve of qRT-PCR is shown in Figure S1A,C. *UBQ-E2-10* was used as the internal reference gene [48], and the reaction volume was 20 μL. The operating parameters of the qRT-PCR were as follows: 95 °C for 30 s, followed by 40 cycles of 95 °C for 10 s and 58 °C for 30 s. The relative expression levels of each gene were calculated using the 2^−ΔΔCt^ method.

### 4.7. Data Analysis

The metabolic data were processed by multivariate statistical analysis, including principal component analysis (PCA), hierarchical cluster analysis (HCA), and orthogonal partial least squares discriminant analysis (OPLS–DA). The OPLS–DA model with variable importance in projection (VIP ≥ 1) threshold and fold change (FC ≥ 2 or FC ≤ 0.5) was used to screen for differential metabolites of *P. cyrtonema*. The different samples were screened for differential metabolites by hierarchical clustering, and the corresponding differential metabolites obtained were submitted to the KEGG (Kyoto Encyclopedia of Genes and Genomes) database for pathway correlation analysis.

In addition, the data were statistically evaluated by one-way ANOVA, least significant difference (LSD) test, and principal component analysis (PCA) using IBM SPSS Statistics 26 software (SPSS, Inc., Chicago, IL, USA). Mean differences with *p* < 0.05 were considered significant. Finally, Bar graphs were created using GraphPad Prism 8 software (GraphPad, San Diego, CA, USA).

## 5. Conclusions

This study constitutes the first comparative analysis of the metabolic compositions of the rhizomes of three germplasms (JL, JZ, and GK) of *P. cyrtonema* and three age sections (AT, BT, and CT) of the GK germplasm by widely targeted metabolomics. The results revealed that the GK germplasm is rich in polysaccharides, alkaloids, and lipids; the JL germplasm has high levels of flavonoids, steroids, and amino acids; and the JZ germplasm contains more phenolic acids. The AT age section was determined to be rich in polysaccharides, steroids, organic acids, and lipids, and more flavonoids, alkaloids, and amino acid metabolites were detected in the CT age section. Additionally, this study identified a total of 41 key differential metabolites, which can be used as markers to distinguish the three *P. cyrtonema* germplasms. Differences in the pentose phosphate pathway (ko00030) and metabolic pathways (ko01100) may be the main causes of the metabolite differences between the age sections. Furthermore, TAG, which is associated with resistance, was found at the highest level in the GK germplasm in this study. The qRT-PCR results suggest that the *PcDGAT1*, *PcDGAT2.4*, *PcGPAT9.1*, *PcLPAT2.9*, and *PcLPAT4.3* genes may play an important role in TAG synthesis in *P. cyrtonema*. The present study reveals differences in metabolite profiles between different germplasms and different age sections of *P. cyrtonema*, and the molecular mechanism of triacylglycerol synthesis was explored, providing a new theoretical reference for the breeding and product development of new varieties of *Polygonatum* species.

## Data Availability

Data is contained within this article and its supplementary material. Raw (unprocessed) data are available on request from the corresponding author.

## Supplementary Materials

The supporting information can be downloaded at: https://www.mdpi.com/article/10.3390/ijms24076077/s1.
