# Supplementary material for: Metabolic Composition and Quality Traits of Polygonatum cyrtonema Hua from Different Germplasms and Age Sections Based on Widely Targeted Metabolomics Analysis
Source: Int J Mol Sci. 2023 Mar 23;24(7):6077. doi: 10.3390/ijms24076077 (PMC10094609; doi:10.3390/ijms24076077)
Supplement: Supplementary file 1 [file ijms-24-06077-s001.zip › ijms-2238195-supplementary.pdf]

Supplementary material

Table S1. qRT-PCR primers in this study.

Gene name		Primer Sequences (5′ -3′)	Annealing temperature (°C)	GC%
DGAT1	Forward	AAAGGTTGGGTAATACGCC	56.6	47.4
	Reverse	GCCAGACATACAAAGTGGG	56.5	52.6
DGAT2.4	Forward	AGAACAGACTGGGCAGGAAA	59.8	50
	Reverse	AGGACCTTGATCTTCGGCA	60.8	52.6
GPAT9.1	Forward	AAGTATCATGGACCTCGCCC	61.2	55
	Reverse	TGGCCACAATTTCACGATC	60.5	47.4
GPAT9.3	Forward	ATCGCCTACACTAACTGAAGC	56.3	47.6
	Reverse	AAACACAATCCACCCAGC	56.8	50
LPAT1.1	Forward	TGGTTGCTAAGGTTTGGG	57.1	50
	Reverse	ATGCCCGTCTTGCTAATG	57.2	50
LPAT2.9	Forward	GCCTCCTGTTTCTTCTTTCG	59.1	50
	Reverse	AGACCTGCCCACCAATCTAC	59	55
LPAT4.3	Forward	ACGAAGACACCAGCCATTG	59.7	52.6
	Reverse	GCCACATAGATAGCCAAGTCC	58.7	52.4
UBQ-E2-10	Forward	GGACCCAGAAGTACGCAATG	60.5	55
	Reverse	AATTACCAGGGATACAGCACC	57.9	47.6

Table S2. Key differential metabolites in three germplasms (JL, JZ, and GK).

Compounds	Class	Relative ionic strength		
		JL	JZ	GK
3-amino-2-naphthoic acid	Alkaloids	1.38E+07	4.30E+06	1.69E+06
3-Indoleacrylic acid*	Alkaloids	1.48E+07	4.94E+06	1.82E+06
Methoxyindoleacetic acid	Alkaloids	2.08E+05	6.46E+04	2.72E+04
N-Cis-Feruloyltyramine	Alkaloids	6.57E+04	2.66E+05	8.94E+05

p-Coumaroyltyramine*	Alkaloids	1.59E+04	1.61E+05	7.64E+05
Tataramide A	Alkaloids	5.95E+03	1.96E+04	8.87E+04
N-trans-p-Coumaroyloctopamine*	Alkaloids	1.06E+04	4.50E+04	3.76E+05
N-Trans-Feruloylphenacylamine	Alkaloids	3.50E+04	1.48E+05	3.95E+05
N-trans-p-Coumaroylphenacylamine	Alkaloids	1.27E+04	7.18E+04	6.00E+05
N-Trans-Feruloyloctopamine*	Alkaloids	6.03E+04	1.75E+05	5.08E+05
N-Feruloyloctopamine	Alkaloids	1.06E+04	2.81E+04	1.09E+05
N-Feruloyl-3-methoxytyramine*	Alkaloids	4.94E+02	1.01E+04	1.19E+05
N-Trans-Feruloyl-3'-O-methyldopamine*	Alkaloids	1.60E+03	8.75E+03	1.11E+05
N-Cis-Feruloyl-3'-O-methyldopamine*	Alkaloids	2.48E+03	9.02E+03	1.04E+05
(2E)-3-(4-Hydroxyphenyl)-N-[2-(4-hydroxyphenyl)ethyl]-2-propenamide*	Alkaloids	1.38E+04	1.50E+05	8.12E+05
(2Z)-N-[2-(3,4-Dihydroxyphenyl)-2-hydroxyethyl]-3-(4-methoxyphenyl)-2-propenamide*	Alkaloids	6.48E+04	1.78E+05	5.25E+05
Luteolin-7-O-glucoside (Cynaroside)*	Flavonoids	2.07E+05	1.59E+04	3.87E+04
6-C-Methylquercetin-3-O-glucoside*	Flavonoids	4.40E+05	4.74E+04	1.93E+04
3'-O-Methyltricetin-7-O-glucoside*	Flavonoids	4.07E+05	3.49E+04	1.64E+04
Gossypetin-3-O-glucoside	Flavonoids	1.18E+05	1.89E+04	9.00E+00
2,6-Dimethoxybenzaldehyde	Phenolic acids	3.62E+04	1.30E+04	4.71E+03
P-Hydroxycinnamic acid p-hydroxyphenethylamine*	Phenolic acids	2.41E+04	1.63E+05	8.39E+05
Salicylic acid-2-O-glucoside*	Phenolic acids	1.22E+05	9.04E+05	3.89E+05
6-O-Acetylarbutin	Phenolic acids	1.02E+04	9.00E+00	4.34E+03
Methoxysalicylic acid glucoside*	Phenolic acids	2.16E+05	1.96E+06	6.78E+05
5-Glucosyloxy-2-Hydroxybenzoic acid methyl ester*	Phenolic acids	1.43E+05	2.99E+05	2.54E+04
3,4,5-Trimethoxyphenyl-1-O-Glucoside	Phenolic acids	4.54E+05	4.04E+06	1.37E+06
Benzyl β-primeveroside*	Phenolic acids	1.23E+05	2.66E+05	5.13E+04
DL-Tryptophan	Amino acids and derivatives	2.94E+07	1.32E+07	5.38E+06
Seryl threonine	Amino acids and derivatives	1.41E+05	4.50E+04	1.86E+04
Threonylleucine	Amino acids and derivatives	1.72E+07	4.15E+06	1.24E+06
L-Valyl-L-Phenylalanine	Amino acids and derivatives	6.24E+03	2.57E+04	5.26E+04
(10E,12Z)-9-Oxooctadeca-10,12-dienoic acid	Lipids	1.13E+03	2.56E+03	1.22E+04
E,E,Z-1,3,12-Nonadecatriene-5,14-diol	Lipids	4.99E+02	1.22E+03	2.04E+04
Eicosadienoic acid	Lipids	1.79E+04	4.35E+04	3.64E+05
2-α-Linolenoyl-glycerol	Lipids	1.44E+04	6.47E+03	9.31E+05
MG 18:3	Lipids	1.36E+04	6.17E+03	9.42E+05
naphthisoxazol A*	Others	1.34E+07	4.38E+06	1.66E+06
Tridecadienoyl sulfate	Others	1.34E+04	2.96E+04	2.77E+03
Ruscogenin-1-O-carboxyglucosyl(1,2)rhamnoside	Steroids	2.69E+05	9.46E+04	1.87E+04
Muconic acid	Organic acids	3.82E+05	1.55E+05	5.21E+04

Note: JL: pointed-leaved green stem, JZ: pointed-leaved purple stem, GK: broad-leaved green stem. "*" indicates an isomer, and the same below.

Table S3. Key differential metabolites in three age sections (AT, BT, and CT).

Compounds	Class	Relative ionic strength		
		AT	BT	CT
LysoPC 20:2*	Lipids	2.42E+05	1.17E+05	5.14E+04
4-Methoxybutyl Acetate	Lipids	7.92E+04	3.81E+04	1.69E+04
LysoPC 20:2(2n isomer)*	Lipids	9.93E+04	4.89E+04	1.88E+04
N-Feruloyltryptamine	Alkaloids	3.19E+04	1.01E+04	9.00E+00
N-(4-hydroxyphenethyl)-glucosamine	Alkaloids	3.17E+05	6.98E+05	2.71E+06
Sibiricose A6	Phenolic acids	7.39E+03	9.00E+00	3.68E+04
3-Hydroxy-5-Methylphenol-1-O-Glucoside	Phenolic acids	1.71E+06	5.33E+05	1.75E+05
Furanofructosyl-α-D-(3-mustard acyl)glucoside	Phenolic acids	4.69E+03	1.57E+04	5.79E+04
L-Serine	Amino acids and derivatives	1.28E+06	3.41E+06	6.88E+06
L-Alanyl-L-Alanine	Amino acids and derivatives	5.66E+04	1.25E+04	2.74E+04
3-Aminoisobutyric acid*	Organic acids	2.95E+04	1.34E+04	3.92E+03
1-Pyrroline-4-hydroxy-2-carboxylic acid	Organic acids	1.79E+05	9.00E+00	4.13E+05
Phloretin	Flavonoids	4.65E+04	3.06E+05	9.65E+05
Diosgenin	Steroids	1.13E+05	1.03E+04	9.00E+00
Trehalose 6-phosphate	Saccharides and Alcohols	3.94E+05	2.59E+06	1.05E+06
Tridecadienoyl sulfate	Others	3.25E+05	1.32E+06	3.09E+06

Note: AT: one-year age sections, BT: two-year age sections, CT: three-year age sections.

Table S4. Relative expression of *GPAT*, *LPAT,* and *DGAT* genes in the transcriptome of different tissue sites of *P. cyrtonema.*

Index	Gene name	Class	F	L	R	S	T
Cluster-11683.26510	PcGPAT1.2		0.00	0.00	0.00	0.00	1.06
Cluster-11683.201308	PcGPAT1.3		1.29	0.13	28.28	0.00	0.45
Cluster-11683.106825	PcGPAT9.2		4.54	8.03	6.21	6.02	3.56
Cluster-11683.56323	PcGPAT6.1		0.99	0.00	2.35	0.04	0.53
Cluster-29692.0	PcGPAT1.5		1.04	0.00	1.15	0.00	1.33
Cluster-11683.178507	PcGPAT9.5		1.20	1.93	0.91	0.97	1.77
Cluster-11683.137258	PcGPAT4	GPAT	83.60	3.58	27.64	2.96	7.26
Cluster-11683.117604	PcGPAT9.3		17.84	11.21	20.53	15.70	12.04
Cluster-11683.145205	PcGPAT3.2		0.20	0.09	1.00	0.00	0.00
Cluster-11683.226019	PcGPAT1.4		0.00	0.00	1.39	0.00	0.02
Cluster-11683.131185	PcGPAT9.4		3.82	4.21	3.26	6.01	1.67
Cluster-11683.76883	PcGPAT6.3		2.04	4.21	0.98	0.82	1.03
Cluster-11683.61945	PcGPAT6.2		0.22	2.57	1.46	1.45	2.14

Cluster-11683.17927	PcGPAT1.1		0.17	0.02	4.67	0.00	0.00
Cluster-11683.60675	PcGPAT5		6.20	0.17	3.55	0.00	0.80
Cluster-11683.49377	PcGPAT3.1		1.39	0.07	3.37	0.37	0.01
Cluster-11683.89039	PcGPAT9.1		18.68	31.84	26.60	32.77	22.48
Cluster-11683.125365	PcATS1.1		27.30	60.12	6.92	50.41	10.00
Cluster-11683.168120	PcATS1.2		0.24	0.77	0.39	0.65	0.95
Cluster-11683.164452	PcLPAT2.7		2.04	2.42	2.24	2.16	1.82
Cluster-11683.140043	PcLPAT2.4		23.98	17.90	16.66	17.50	17.38
Cluster-11683.122314	PcLPAT2.3		14.78	13.09	12.89	16.48	16.93
Cluster-11683.86126	PcLPAT2.1		0.61	1.09	0.81	1.02	0.44
Cluster-11683.105098	PcLPAT2.2		39.50	17.02	13.34	20.85	23.36
Cluster-11683.145946	PcLPAT1.2		67.18	77.56	2.65	33.24	4.43
Cluster-11683.143957	PcLPAT2.5	LPAT	1.02	0.55	0.07	1.13	0.46
Cluster-11683.187439	PcLPAT2.9		15.24	14.57	14.63	12.41	24.34
Cluster-11683.63640	PcLPAT4.1		8.72	22.37	14.10	26.12	11.41
Cluster-11683.116975	PcLPAT1.1		6.14	9.96	7.41	9.10	12.05
Cluster-11683.144893	PcLPAT4.3		12.81	7.21	11.27	9.63	13.23
Cluster-11683.169863	PcLPAT2.8		2.13	2.33	1.16	2.12	0.30
Cluster-11683.95330	PcLPAT4.2		0.20	0.73	0.00	0.20	0.10
Cluster-11683.158348	PcLPAT2.6						
Cluster-11683.114805	PcDGAT1		11.30	13.73	16.87	17.36	14.61
Cluster-11683.125465	PcDGAT2.2		8.36	11.50	6.17	9.70	6.47
Cluster-11683.128175	PcDGAT2.3		19.39	12.13	0.00	58.36	0.05
Cluster-11683.137374	PcDGAT2.4		242.85	295.00	112.25	352.44	160.04
Cluster-11683.139297	PcDGAT2.5		0.21	0.47	0.64	0.29	0.03
Cluster-11683.144767	PcDGAT2.6		1.24	0.81	0.16	0.97	0.57
Cluster-11683.150005	PcWSD1.3	DGAT	23.53	91.68	32.73	38.69	10.95
Cluster-11683.160378	PcWSD1.4		0.89	3.21	0.40	2.93	0.29
Cluster-11683.202350	PcWSD1.5		1.90	0.06	0.35	0.02	0.33
Cluster-11683.61124	PcWSD1.1		1.95	0.62	0.57	0.12	5.80
Cluster-11683.64305	PcWSD1.2		1.60	0.09	0.20	0.00	0.84
Cluster-11683.123837	PcDGAT2.1						
Cluster-11683.175971	PcDGAT2.7						

Note: F: fruit, L: leaf, R: root, S: stem, T: rhizome.

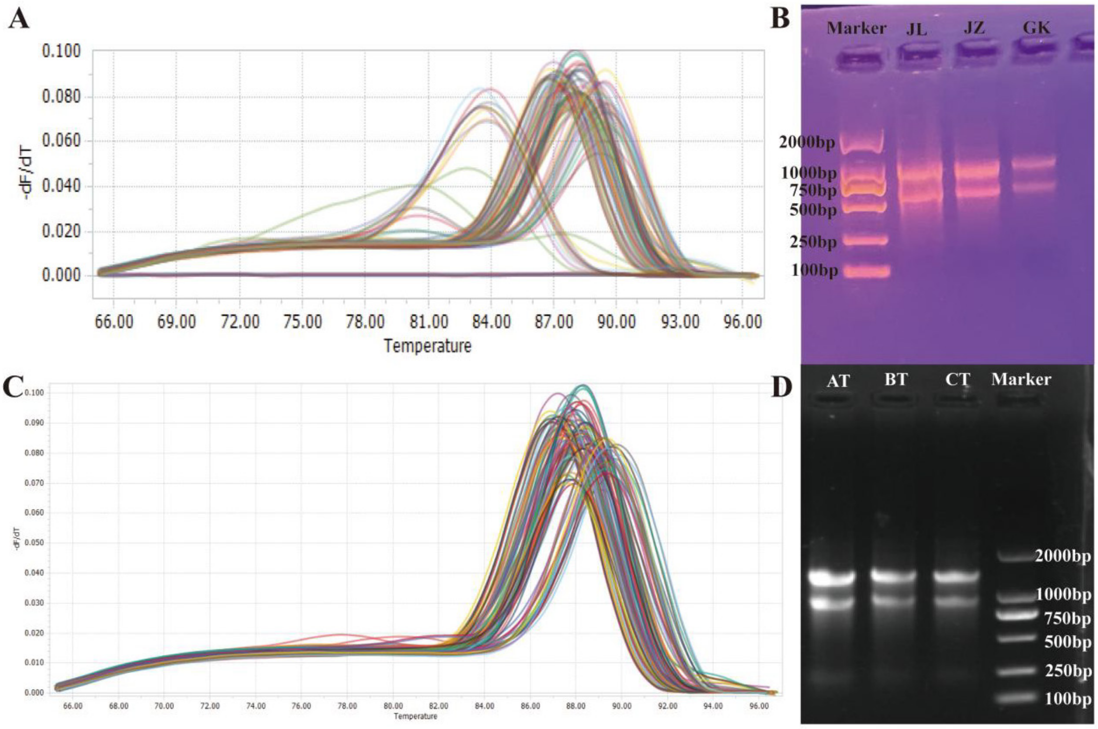

Figure S1. Melting curve (A: different germplasms, C: different age sections) of qRT–PCR and electrophoresis map (B: different germplasms, D: different age sections) of the total RNA isolated from *P. cyrtonema.*

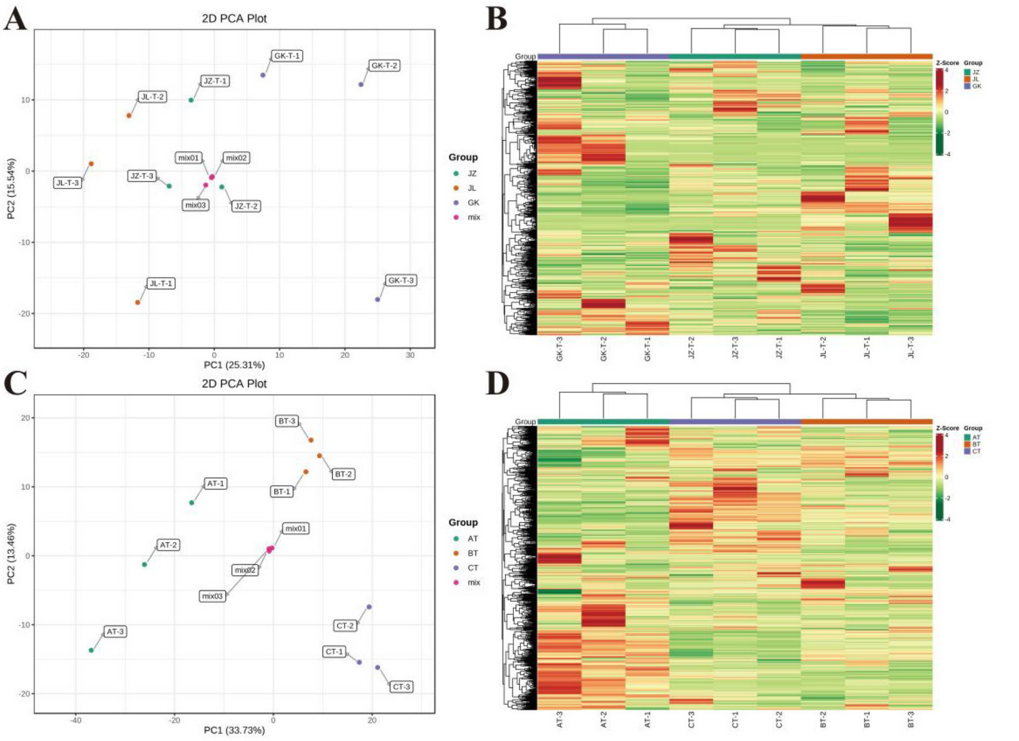

Figure S2. PCA plots (A: different germplasms, C: different age sections) and clustered heat maps (B: different germplasms, D: different age sections) of *P. cyrtonema.*

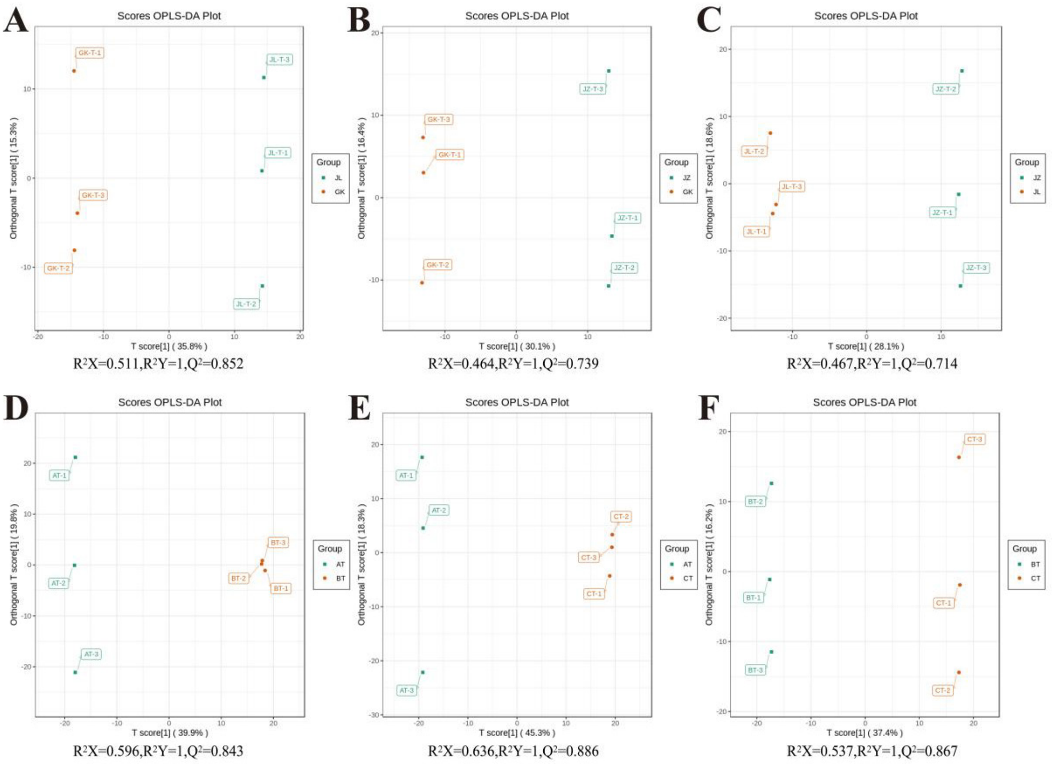

Figure S3. OPLS-DA score plot for *P. cyrtonema* (A: JL vs GK, B: JZ vs GK, C: JZ vs JL, D: AT vs BT, E: AT vs CT, F: BT vs CT).
